# Suprarenal Extract

**Published:** 1901-04

**Authors:** 


### SUPRARENAL EXTRACT.

ANOTHER favorable report is made upon the action of the suprarenal extract in diseases of the respiratory tract. P. Floersheim, in a recent issue of the *Medical Record*, says he used it in rhinitis, acute tracheobronchitis, chronic bronchitis, congestion and edema of the lungs, hemoptysis and pulmonary tuberculosis. Its action in diseases of the upper tubes is more certain and the sensations of dryness, tightness and rawness are relieved, but acute bronchitis when treated from the onset was usually cured in twenty-four hours. The beneficial action obtained in congestion and edema of the lungs was undoubtedly due, not only to the constricting action upon the bloodvessels, but also to the stimulating effect upon the heart. Apparently good results were obtained in cases of hemoptyses, but it is, of course, speculative how much benefit was due to this drug. The powder is administered in three grain capsules, chewed without water and swallowed in a few minutes. When the doses are sufficiently large the action is apparent in from two to fifteen minutes. In some cases the action of the drug is permanent, but in the majority of cases it lasts only from ten minutes to an hour and must be frequently repeated.

